# Decomposition and Decoupling Analysis of CO_2_ Emissions Based on LMDI and Two-Dimensional Decoupling Model in Gansu Province, China

**DOI:** 10.3390/ijerph18116013

**Published:** 2021-06-03

**Authors:** Lele Xin, Junsong Jia, Wenhui Hu, Huiqing Zeng, Chundi Chen, Bo Wu

**Affiliations:** 1 Key Laboratory of Poyang Lake Wetland and Watershed Research, Ministry of Education/School of Geography and Environment, Jiangxi Normal University, Nanchang 330022, China; lelexin513@jxnu.edu.cn (L.X.); wubo@jxnu.edu.cn (B.W.); 2 Centre de Recherche Sur Madagascar, Foreign Languages College, Jiangxi Normal University, Jiangxi Normal University, Nanchang 330022, China; 004654@jxnu.edu.cn; 3 School of Resources Environmental and Chemical Engineering, Nanchang University, Nanchang 330031, China; zenghuiqing@ncu.edu.cn; 4 School of Architecture and Urban Planning, Tongji University, Shanghai 200092, China; chundichen@tongji.edu.cn

**Keywords:** CO_2_ emissions, LMDI, two-dimensional decoupling model, energy consumption, Gansu province

## Abstract

Currently, little attention has been paid to reducing carbon dioxide (CO_2_) emissions of Gansu, and the two-dimensional decoupling model has been rarely used to study the relationship between the economic development and CO_2_ emissions, especially in western China (e.g., Gansu). Thus, here, we first used the Logarithmic Mean Divisia Index (LMDI) to decompose the driving factors of Gansu’s CO_2_ emissions between 2000–2017 and then analyzed the decoupling relationship by using the two-dimensional model. Results showed: (1) Gansu’s CO_2_ emissions increased from 7805.70 × 10^4^ t in 2000 to 19,896.05 × 10^4^ t in 2017. The secondary industry accounted for the largest proportion in Gansu’s CO_2_ emissions, followed by the tertiary industry and the primary industry. (2) The economic output showed the dominant driving effect on Gansu’s CO_2_ emissions growth with the cumulative contribution rate of 201.94%, followed by the effects of industrial structure, population size, and energy structure, and their cumulative contribution rates were 9.68%, 7.81%, and 3.05%, respectively. In contrast, the energy intensity effect presented the most obvious mitigating effect with the cumulative contribution rate of −122.49%. (3) The Environmental Kuznets Curve (EKC) between CO_2_ emissions and economic growth was demonstrated the inverted U-shape in Gansu. The two-dimensional decoupling status was the low level-weak decoupling (WD-LE) during 2000–2017. Thus, dropping the proportion of the secondary industry, reducing the use of carbon-intensive fuel like coal, introducing advanced technologies, and increasing the investment of new energy might effectively restrain the growth of Gansu’s CO_2_ emissions.

## 1. Introduction

Global warming, which is caused by excessive greenhouse gas emissions, has been increasingly recognized as the primary environmental threat to the survival of human beings. Burning fossil fuels such as coal, gas, and oil have produced more than 80% of the world’s energy and more than 90% of global carbon dioxide (CO_2_) emissions [1]. Over 156 years ago, Jevons expressed fears that economic progress in Great Britain would be reversed because the country would run out of coal [2,3]. China also should pay attention to Jevons’ fears. China is currently the largest developing country in the world, and the rapid development of its economy is accompanied by the increase of fossil fuels. Like many other countries, the primary cause of China’s anthropogenic CO_2_ emissions is energy-related fossil fuels combustion [4]. China’s CO_2_ emissions caused by fossil fuel consumption had reached 60.52 × 10^8^ t in 2007, which made China become the world’s biggest carbon emitter [5]. Such a large quantity of CO_2_ emissions has put enormous pressure on reducing its discharge on the Chinese government. Therefore, facing the increase of environmental pressure, China has proposed the strong targets of corresponding independent contribution under the Paris Agreement. Namely, the CO_2_ emissions intensity of per-unit gross domestic product (GDP) in 2030 will be reduced by 60–65% compared with that in 2005, and the proportion of non-fossil fuel will rise to about 20% in primary energy consumption [6]. Furthermore, during the 75th Session of United Nations General Assembly, the Chinese government has proposed to enhance the national independent contributions by adopting more effective policies. Meanwhile, China has come up with a plan to achieve carbon peak by 2030 and carbon neutrality by 2060 [7]. Thus, to accomplish the above ambitious targets of emission reduction requires finding efficient ways to control CO_2_ emissions. The provinces of China are special administrative divisions connecting the nation as a whole and the cities, and so they play an important role in realizing the reduction target of China’s CO_2_ emissions [8]. It stands to reason that it is crucial for us to analyze the influencing factors of CO_2_ emissions and accelerate the decoupling process between CO_2_ emissions and economic growth in Gansu province.

Industrial production, service industry, trade, construction, transportation, urbanization, and many other sectors profoundly count on energy consumption [9]. In recent years, the implementations of the Silk Road Economic Belt and the Western Development Strategy have provided more opportunities for developing the economy in Gansu province. Thus, the industrialization and urbanization have been rapidly enhanced, and the economy has been developed conspicuously in Gansu. To be specific, the GDP per capita rose from 0.05 × 10^4^ United States dollars (USD) in 2000 to 0.34 × 10^4^ USD in 2019, its annual average growth rate being 10.59%. Meanwhile, the urbanization rate increased from 24.01% in 2000 to 46.39% in 2017 [10]. Hence, the level of economic development in Gansu might stand to maintain a development trend at a relatively fast growth rate in the coming period. However, with the rapid development of its economy, Gansu has also been counting significantly on energy consumption. Specifically, the energy consumption of Gansu increased from 3186.51 × 10^4^ tons of standard coal equivalent (tsce) in 2000 to 7806.49 × 10^4^ tsce in 2017 [10], its growth rate being 144.99%. Thus, the research of the existing decoupling status between Gansu’s CO_2_ emissions and its economic growth seems to be of equally great importance.

As a major energy resource province of China, Gansu also takes on important responsibilities in realizing China’s CO_2_ reduction. Therefore, the targets of independent emission reduction were proposed to control greenhouse gas emissions in Gansu during the “13th Five-Year Plan”. Gansu is one of the most important provinces of CO_2_ emissions reduction in western China regions. If the Chinese government wants to reduce CO_2_ emissions, Gansu must be a very important province to be taken into consideration. However, intense environmental pressure, relatively fragile environmental conditions, and the heavy and low-value-added industrial base are the main challenges of Gansu province [11]. Thus, faced with the above challenges, it is necessary and vital to coordinate the economic growth and CO_2_ emissions in Gansu. Furthermore, the objectives of this paper are therefore to focus on the provincial level (Gansu) by considering both factors influencing CO_2_ emissions and the two-dimensional decoupling status of CO_2_ emissions from its economic development level. We expect that this study might provide some significant insights and references for researching CO_2_ emissions at the provincial level in both China and other developing countries.

This research innovates and contributes to the existing works in two areas. First, it decomposes changes in CO_2_ emissions of Gansu’s three major economic sectors and quantifies the contribution of five factors during 2000–2017, so as to provide helpful information for relevant policymakers. Second, it analyzes the decoupling relationship by using the two-dimensional decoupling model, which can help Gansu province accelerate the decoupling process. Exploring the two-dimensional decoupling relationship between CO_2_ emissions and economic development in Gansu province is forward-looking and representative for the provincial level in China. Therefore, our study stands to enrich the status quo of its academic endeavor.

The rest of the paper is arranged as follows: Section 2 reviews the related literature. Section 3 describes the data sources and introduces the methodology adopted in the study. Section 4 devotes to the empirical results and discussion. Section 5 provides the main conclusions, corresponding policy implications, and limitations.

## 2. Literature Review

### 2.1. CO_2_ Emissions Decomposition Analysis Methods

Generally, there are two decomposition methods commonly used in the study on drivers of CO_2_ emissions, namely, structural decomposition analysis (SDA) and index decomposition analysis (IDA) [12]. SDA is generally supported by the data of input–output table and has a large time span, which is not conducive to in-depth research. IDA is based on the aggregate data of each industry, which is more suitable for time-series analysis [13]. The Logarithmic Mean Divisia Index (LMDI) has become the most popular method among IDA because it has no unexplainable residuals, and it can handle problems of zero value and negative value [14]. Thus, the LMDI method has been extensively used in the decomposition analysis of CO_2_ emissions changes.

In recent years, an increasing number of researchers have used the LMDI to study China’s CO_2_ emissions [15,16,17]. Their studies had discovered that the effect of economic output played the dominant role in increasing CO_2_ emissions, while energy intensity presented the most obvious mitigating effect. Feng et al. [18] analyzed the factors of CO_2_ emissions in the United States from 1997 to 2013. The results indicated that rising emissions were primarily driven by economic growth before 2007. After 2007, decreasing emissions were largely a result of economic recession with changes in fuel mix (for example, substitution of natural gas for coal) playing a comparatively minor role. Ortega-Ruiz et al. [19] explored the main drivers of CO_2_ emissions in India during 1990–2016. The results showed that India’s CO_2_ emissions increased by 276% because of the rapid economic growth, while the energy intensity has been the main factor reducing CO_2_ emissions by approximately −47%. Román-Collado et al. [20] found that the key driving factors were economic activities and population effects in Latin America, followed by fossil fuels and carbonization effects, while intensity effect was an inhibitory factor. Furthermore, some researchers analyzed the drivers of CO_2_ emissions from aspects of energy structure, population size, energy intensity, economic growth, and other factors in Eastern and central China, Yangtze River Delta, and Beijing–Tianjin–Hebei Region, respectively [21,22,23]. The above studies mainly focused on countries or large-scale areas and achieved good results by analyzing the factors of CO_2_ emissions. In addition, LMDI model has also been widely used to analyze the drivers of CO_2_ emissions in various industry sectors [24,25,26,27]. At the same time, many scholars have applied the LMDI model to researching the drivers of CO_2_ emissions at the provincial level, Specifically Jiangxi, Jilin, and Liaoning, respectively [28,29,30]. Jia et al. [28] have decomposed the energy-related industrial carbon emissions (ERICE) from both the macroeconomic and the microeconomic scales. The results showed that output, research and development (R&D) intensity, energy structure, and investment intensity were mainly responsible for the increase of the ERICE. Inversely, the R&D efficiency, energy intensity, and industrial structure presented obvious mitigating effects on the ERICE. As mentioned above, LMDI method estimated the quantitative impact on each driving factor of CO_2_ emissions. Few existing works have utilized this method to study the driving factors of Gansu’s CO_2_ emissions changes, especially their degrees of effect on the three major industries. Therefore, this paper employed the LMDI method to investigate the driving factors of CO_2_ emissions in Gansu province.

### 2.2. Decoupling Analysis Methods

With the aggravation of global warming and environmental degradation, the strategies of sustainable development have been pursued in many countries. Therefore, many scholars have concentrated on researching the decoupling relationship between CO_2_ emissions and economic development. Generally, the Tapio decoupling model and the Organization for Economic Cooperation and Development (OECD) decoupling model have become the main fundamental theories. However, the OECD decoupling model has some limitations and shortcomings. Specifically, the indicator is easily influenced by the decoupling elasticity and lacks explicit criteria [31]. The Tapio decoupling model refined the type of decoupling and solved the problem of basic period selection [32], which has become a theoretical foundation for many decoupling analyses. For example, some scholars analyzed the decoupling relationship in Jiangsu [33], Beijing [34], and Xinjiang [35] by using the Tapio decoupling model. Moreover, Wang et al. [36] analyzed the decoupling relationship between CO_2_ emissions and economic growth in China. The results found that the CO_2_ emissions of all regions showed a stable decoupling trend, and the decoupling status also indicated that most regions were still in the status of expansive coupling or weak decoupling by the end of 2016. However, Song et al. [37] thought that the Tapio decoupling model only described what kind of decoupling status occurred and could not distinguish the decoupling status of different economic development levels. Therefore, based on Environmental Kuznets Curve (EKC) hypothesis and Tapio decoupling model, the two-dimensional decoupling model between CO_2_ emissions and economic development was established to research the decoupling relationship. As the model was used to analyze the decoupling status of different economic development levels, we employed two-dimensional decoupling model to reveal the decoupling relationship between CO_2_ emissions and economic growth in Gansu.

### 2.3. Research on CO_2_ Emissions in Gansu Province

**Figure 1:**
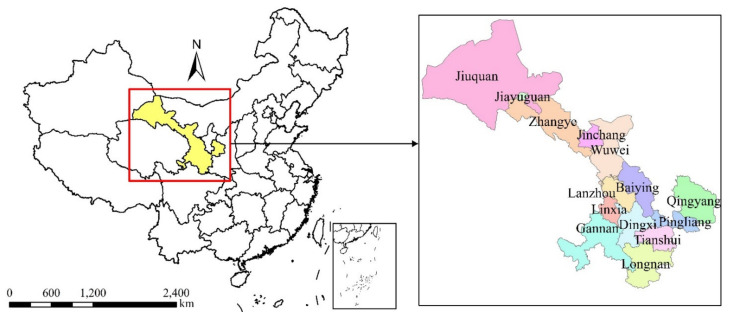
Geographical location of Gansu province.

Gansu, located in the Northwest China mainland (Figure 1), between latitude 32°11′–42°57′ N and longitude 92°13′–108°46′ E, is one of the important industrial bases which have abundant energy resources in China. At present, few studies have analyzed the drivers of CO_2_ emissions and decoupling relationship between CO_2_ emissions and economic growth in Gansu [38,39,40,41,42]. Qi et al. [40] applied LMDI to setting up the decomposition model of per capita CO_2_ emissions. The results revealed that economic growth was a crucial factor in speeding up the increase of Gansu’s per capita CO_2_ emissions. However, the energy intensity was the main factor in controlling CO_2_ emissions, and energy structure has no obvious influence on Gansu’s per capita CO_2_ emissions. Zhang et al. [41] analyzed the corresponding factors of Gansu’s CO_2_ emissions by using the Stochastic Impacts by Regression on Population, Affluence, and Technology (STIRPAT) model from 1997 to 2008 and found that population growth and economic development had a great impact on CO_2_ emissions growth, at the same time, the improvement of living standards had further accelerated CO_2_ emissions growth. Ahmad et al. [42] examined the impact of tourism on the environmental pollution of five provinces located in the heart of One Belt One Road over the period of 1991–2016. The investigating results of Gansu province indicated that the tourism related activities boosted CO_2_ emissions and they had a significantly negative impact on the environment. In a word, the existing studies neglected the connection between CO_2_ emissions and economic growth and treated the three different industries as a whole. Environmental protection, however, is an inclusive concept which should include wise adjustment in sub-industries to mitigate the increasing of CO_2_ emissions in Gansu.

Therefore, from the research and discussions above, we can conclude that to date, many scholars have researched CO_2_ emissions from a large-scale perspective in whole countries or certain regions. In China, researchers have often focused on studying the developed eastern regions. However, underdeveloped regions, especially those in western China, such as Gansu Province, have not received enough attention, and few findings can be found in the existing literature around the world. In a brief, some studies have calculated the total of Gansu’s CO_2_ emissions at different time-series and used different methods to analyze the CO_2_ emissions. The studies had a lack of systematic analysis of CO_2_ emissions of the three different industries in Gansu, therefore, in order to fill such a gap, in this paper we systematically analyzed the drivers of Gansu’s CO_2_ emissions in three different industries and then analyzed the two-dimensional decoupling relationship in Gansu from 2000 to 2017. We firmly believe this study enjoys some innovative significance in helping Gansu province make mitigation policies to achieve low-carbon development.

## 3. Data and Methodology

### 3.1. Data Description

The source of energy consumption is divided into three different industries, namely the primary, secondary, and tertiary industries. The primary industry refers to agriculture, forestry, animal husbandry, fishery, and water conservancy. The secondary industry refers to industry and construction. The tertiary industry refers to transportation, storage, postal and telecommunications services, wholesale, retail trade and catering services, and others. The fuel types of energy consumption include raw coal, coke, crude oil, gasoline, kerosene, diesel oil, fuel oil, liquefied petroleum gas, and natural gas. Gansu was a net export province of electricity and did not emit indirect CO_2_ emissions from 2000–2017. In addition, electricity is not the primary energy source [31], so the CO_2_ emissions of electricity will not be calculated in this paper. The data, including the relevant GDP and various energy consumption of Gansu, are derived from the Gansu Development Yearbook (2000–2017) [10]. At the same time, in order to eliminate the influence of price fluctuation, we deflated the economic output at the current prices to constant 2000 prices using the corresponding price indices. The relevant GDP was converted into USD based on the average annual exchange rate of each year.

### 3.2. Accounting Method of CO_2_ Emissions

There are no direct monitoring data of CO_2_ emissions. Thus, most of the CO_2_ emissions research has been informed by estimating energy consumption. In this paper, we adopted the method provided by the Intergovernmental Panel on Climate Change (IPCC) [43], and the CO_2_ emissions of energy consumption can be calculated by the following equation:

(1) $$C=\sum_{i=1}^{3}\sum_{j=1}^{9}C_{ij}=\sum_{i=1}^{3}\sum_{j=1}^{9}E_{ij}\times f_{j}$$

where C denotes the CO_2_ emissions of energy consumption, i denotes the three different industries; j denotes the fuel types of energy consumption; $E_{ij}$ denotes the consumption of energy j by industries i; $f_{j}$ denotes the CO_2_ emissions coefficients of j fuel types, and it is estimated according to the carbon content, carbon oxidation rate, and net calorific value provided by IPCC. The calculation results of CO_2_ emissions coefficients of different energy types were listed in Table 1.

### 3.3. Decomposition Model of Drivers

According to the expanded Kaya identity and LMDI model [44], the total of Gansu’s CO_2_ emissions of energy consumption can be expressed as the following equation:

(2) $$C=\sum_{i}\sum_{j}C_{ij}=\sum_{i}\sum_{j}\frac{C_{ij}}{E_{ij}}\times \frac{E_{ij}}{E_{i}}\times \frac{E_{i}}{G_{i}}\times \frac{G_{i}}{G}\times \frac{G}{P}\times P=\sum_{i}\sum_{j}F_{ij}\times S_{ij}\times I_{i}\times D_{i}\times R\times P$$

where the variables are defined in Table 2.

In order to quantitatively analyze the influencing factors in the total CO_2_ emissions, the additive LMDI method was used to decompose Equation (2). The changes of total CO_2_ emissions from the base year (*C*_0_) to the target year ($C_{t}$) can be decomposed as the following six effects:

(3) $$\Delta C_{tot}=\Delta C_{t}-\Delta C_{0}=\Delta C_{F}+\Delta C_{ES}+\Delta C_{EI}+\Delta C_{IS}+\Delta C_{G}+\Delta C_{P}$$

The above-mentioned six effects can be written as the follows:

(4) $$\Delta C_{F}=\sum_{i}\sum_{j}\frac{C_{ij}^{t}-C_{ij}^{0}}{\ln C_{ij}^{t}-\ln C_{ij}^{0}}\times \ln\frac{F_{ij}^{t}}{F_{ij}^{0}}$$

(5) $$\Delta C_{ES}=\sum_{i}\sum_{j}\frac{C_{ij}^{t}-C_{ij}^{0}}{\ln C_{ij}^{t}-\ln C_{ij}^{0}}\times \ln\frac{S_{ij}^{t}}{S_{ij}^{0}}$$

(6) $$\Delta C_{EI}=\sum_{i}\sum_{j}\frac{C_{ij}^{t}-C_{ij}^{0}}{\ln C_{ij}^{t}-\ln C_{ij}^{0}}\times \ln\frac{I_{i}^{t}}{I_{i}^{0}}$$

(7) $$\Delta C_{IS}=\sum_{i}\sum_{j}\frac{C_{ij}^{t}-C_{ij}^{0}}{\ln C_{ij}^{t}-\ln C_{ij}^{0}}\times \ln\frac{D_{i}^{t}}{D_{i}^{0}}$$

(8) $$\Delta C_{G}=\sum_{i}\sum_{j}\frac{C_{ij}^{t}-C_{ij}^{0}}{\ln C_{ij}^{t}-\ln C_{ij}^{0}}\times \ln\frac{R^{t}}{R^{0}}$$

(9) $$\Delta C_{P}=\sum_{i}\sum_{j}\frac{C_{ij}^{t}-C_{ij}^{0}}{\ln C_{ij}^{t}-\ln C_{ij}^{0}}\times \ln\frac{P^{t}}{P^{0}}$$

(10) $$L(C_{ij}^{t}\;,C_{ij}^{0})=\left\{\begin{array}{c} \frac{C_{ij}^{t}-C_{ij}^{0}}{\ln C_{ij}^{t}-\ln C_{ij}^{0}},\\ C_{ij}^{t}\;or\;C_{ij}^{0}\;, \end{array}\right.\begin{array}{c} C_{ij}^{t}\neq C_{ij}^{0}\\ C_{ij}^{t}=C_{ij}^{0} \end{array}$$

It is noteworthy that the CO_2_ emissions coefficients of various fuels are all assumed to be fixed when calculating Gansu’s CO_2_ emissions, which has no contributions to the changes of CO_2_ emissions. That is to say, the $\Delta C_{F}$ in Equation (3) should be 0 [22]. Thus, the changes of CO_2_ emissions can be divided into five effects: the energy structure effect ($\Delta C_{ES}$), the energy intensity effect ($\Delta C_{EI}$), the industrial structure effect ($\Delta C_{IS}$), the economic output effect ($\Delta C_{G}$), and the population scale effect ($\Delta C_{P}$). All five effects will be used to analyze their relative impacts on CO_2_ emissions during 2000–2017 in Gansu.

### 3.4. Two-Dimensional Decoupling Model

#### 3.4.1. EKC Hypothesis

The EKC hypothesis was first proposed by Grossman and Krueger [45,46] to describe the inverted U-shape curve relationship between environmental pollution and the level of GDP per capita. According to Xia et al. [47], the EKC curve can be estimated by the following formula:

(11) $$C_{t}=\alpha_{0}+\alpha_{1}g^{t}+\alpha_{2}{\left(g^{t}\right)}^{2}$$

where $C_{t}$ denotes the per capita CO_2_ emissions in year t; $g^{t}$ denotes the GDP per capita in year t; $\alpha_{0}$ is the constant term, $\alpha_{1}$ is the first-order coefficients; and $\alpha_{2}$ is the second-order coefficients. If $\alpha_{1}$ > 0, $\alpha_{2}$ < 0, the EKC curve satisfies the inverted U-shape.

#### 3.4.2. Tapio Decoupling Model

Decoupling analysis is the common method to research the relationship between economic growth and environmental pressure. As the Tapio approach has the advantages of simple calculation process and clear judgement standard, it has become one of the most popular decoupling approaches in energy economics since it was proposed [48]. Based on the Tapio decoupling model [32], the decoupling index between CO_2_ emissions and economic growth is expressed by the following formula:

(12) $$D=\frac{\Delta C/C}{\Delta GDP/GDP}$$

where D denotes the decoupling indicators; ΔC and ΔGDP denote the changes of CO_2_ emissions and GDP, respectively. The decoupling states were divided into 8 types by Tapio, as shown in Table 3.

#### 3.4.3. Two-Dimensional Decoupling Model

**Figure 2:**
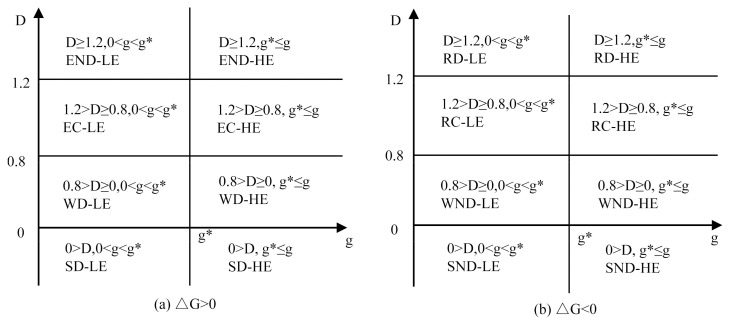
The divisions of decoupling status of the two-dimensional decoupling model. (**a**) ΔG > 0, (**b**) ΔG < 0 (g* *means* the threshold value of per capita GDP).

According to Song et al. [37], based on the critical point and Tapio decoupling criteria, the two-dimensional decoupling standard is shown in Figure 2. The left side of g* (the threshold value of per capita GDP) represents the stages with low level of economic development (LE), while the right side of g* represents the stages with high level of economic development (HE), a model that can distinguish the influence of economic development levels upon the decoupling status in different periods of the economic development.

## 4. Results and Discussion

### 4.1. Overview of Energy Consumption and CO_2_ Emissions in Gansu

**Figure 3:**
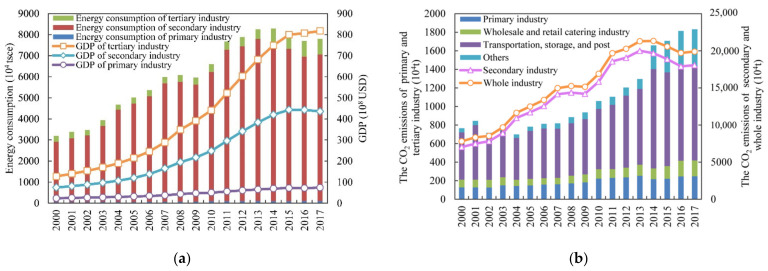
The changes of energy consumption and GDP in three different industries (**a**), and the CO_2_ emissions of each industry in Gansu from 2000 to 2017 (**b**).

The Western Development Strategy has been implemented since 2000, Gansu’s GDP has rapidly grown from 127.18 × 10^8^ USD in 2000 to 812.63 × 10^8^ USD in 2017 (Figure 3a), and its average annual growth rate was 10.27%. In terms of GDP, the secondary and tertiary industries accounted for the lion’s share, while the primary industry accounted for a relatively small portion (Figure 3a). With rapid economic growth, energy consumption increased from 3186.51 × 10^4^ tsce in 2000 to 7806.49 × 10^4^ tsce in 2017, with an average annual growth rate of 5.44% (Figure 3a). Meanwhile, the secondary industry is the largest source of total energy consumption, followed by the tertiary industry and then by the primary industry. With the rapid increase of energy consumption, CO_2_ emissions of the whole industry increased from 7805.70 × 10^4^ t in 2000 to 19,896.05 × 10^4^ t in 2017 (Figure 3b), with an average annual growth rate of 5.66%. The general trend of CO_2_ emissions of the whole industry could be subdivided into two phases: a rapid increase phase (2000–2014) and a fluctuating decline phase (2014–2017). In the first stage, the CO_2_ emissions increased from 7805.70 × 10^4^ t in 2000 to 21,326.87 × 10^4^ t in 2014, with an average annual growth rate of 7.44%. This might be mainly attributed to the proportion of carbon-intensive fuels used like coal, which grew from 48.22% in 2000 to 62.98% in 2014. In the second stage, the CO_2_ emissions decreased from 21,326.87 × 10^4^ t in 2014 to 19,896.05 × 10^4^ t in 2017, with an average annual decline rate of 2.29%. The main reason was that the proportion of coal decreased to 60.88% in 2017. In the meantime, the energy efficiency has been improved and the use of fossil energy has been optimized in Gansu. Moreover, it was obvious that the average annual growth rate of GDP was higher than that of energy consumption and CO_2_ emissions between 2000–2017.

Different sub-industries accounted for different proportions in the total CO_2_ emissions. The CO_2_ emissions of the secondary industry increased by 11,022.33 × 10^4^ t during 2000–2017 (Figure 3b), with an average annual growth rate of 5.70%. It was followed by the tertiary industry and the primary industry, which increased by 948.39 × 10^4^ t and 119.63 × 10^4^ t (Figure 3b), with its average annual growth rates of 5.51% and 3.97% in the order given. The secondary industry accounted for 92.88% in CO_2_ emissions of the whole industry, while the tertiary industry and the primary industry accounted for merely 5.90% and 1.21%, respectively. Besides, it was obvious that the secondary industry was the largest source of CO_2_ emissions, which accounted for more than 90% in the total CO_2_ emissions. The findings in this study were similar to the results of Yang et al. [15], which stated that secondary industry had always dominated the CO_2_ emissions and its proportion of CO_2_ emissions to total CO_2_ emissions had risen from 90.47% to 91.45%. Thus far, as CO_2_ emissions are concerned, the sub-industries of transportation, storage, and the postal service were the largest sectors in the tertiary industry (Figure 3b). Therefore, the secondary industry will be a key sector of emission reduction in the foreseeable future. Meanwhile, the increasing trend of CO_2_ emissions in the tertiary industry ought not to be ignored, especially in the sub-industries of transportation, storage, and the post. These results were consistent with Zhang et al. [49], which showed that the industrial CO_2_ emissions accounted for about 66.3–72.0% of the total CO_2_ emissions and the CO_2_ emissions of the transportation sector were increasing at a high rate.

### 4.2. Decomposition Analysis of CO_2_ Emissions in Gansu

**Figure 4:**
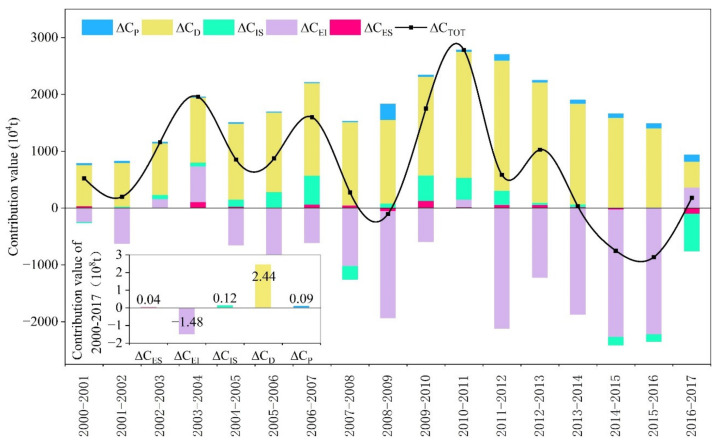
Additive decomposition results of Gansu’s CO_2_ emissions changes from 2000 to 2017 and the entire period. Here, $\Delta C_{TOT}$ means the total CO_2_ emissions changes over time; $\Delta C_{P}$, $\Delta C_{D}$, $\Delta C_{IS}$, $\Delta C_{EI}$, and $\Delta C_{ES}$ indicate the CO_2_ emissions changes caused by population, economic output, industrial structure, energy intensity, and energy structure, respectively.

According to the formula (5–9), the five categories of effects were calculated as shown in Figure 4. To look at the breakdown of contributions to the CO_2_ emissions growth in Gansu, ranked from high to low, the driving factors were the economic output effect (2.44 × 10^8^ t), industrial structure effect (0.12 × 10^8^ t), population size effect (0.09 × 10^8^ t), and energy structure effect (0.04 × 10^8^ t), while the mitigating factor was the energy intensity effect (−1.48 × 10^8^ t) during 2000–2017. These findings were in line with Wang et al. [50], concluding that the economic output effect was the dominant positive driver of the changes in China’s CO_2_ emissions, followed by the effects of industrial structure, population size, and energy structure. Whereas the energy intensity effect was the dominant negative driver.

#### 4.2.1. Energy Intensity

**Figure 5:**
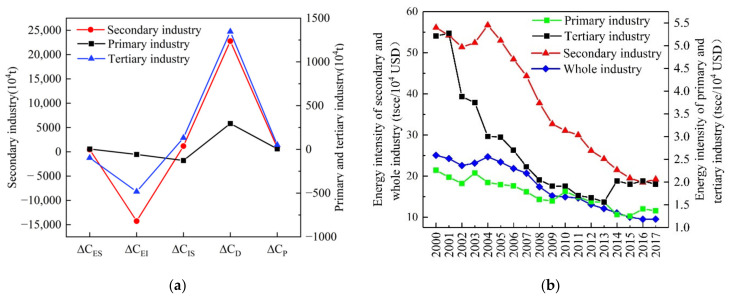
(**a**) The cumulative contribution values of different effects in each industry during 2000–2017 and (**b**)the energy intensity of various industries in Gansu.

Energy intensity is the ratio of energy consumption to GDP and is an important indicator reflecting the energy efficiency of certain regions [27]. The additive decomposition effect of energy intensity was −1.48 × 10^8^ t (Figure 4) in Gansu from 2000–2017, with the corresponding contribution rate of −122.49%. The results showed that the energy intensity was the most important mitigation effect, which was consistent with the previous studies in China [51,52]. This mitigation effect may be caused by the downward trend of energy intensity throughout the whole industry in Gansu. The energy intensity of the whole industry has dropped from 25.05 tsce/10^4^ USD in 2000 to 9.55 tsce/10^4^ USD in 2017 (Figure 5b), with its average annual decline rate of 4.40%. These results suggest that the energy efficiency has been improved in Gansu. However, the energy intensity of Gansu still has a disparity when compares with the developed eastern provinces, and there is also great potential for further reduction. Therefore, the technical exchange should be strengthened with eastern developed provinces and the backward technology should be eliminated in Gansu to mitigate the growth trend of CO_2_ emissions.

The cumulative contribution values of energy intensity in the three different industries were −58.04 × 10^4^ t, −14,267.78 × 10^4^ t, and −483.21 × 10^4^ t, respectively (Figure 5a). The mitigation effect of the secondary industry was most conspicuous and accounted for 96% in the effect of energy intensity, followed by the tertiary industry and the primary industry. This indicated that the mitigation effect of energy intensity was mainly due to the improvement of energy efficiency in the secondary industry. In addition, the energy intensity of each industry showed the mitigation effects because they presented a declining trend during 2000–2017. As shown in Figure 5b, the energy intensity of secondary industry has declined from 56.11 tsce/10^4^ USD in 2000 to 19.26 tsce/10^4^ USD in 2017, with an average annual decline rate of 4.97%. It was followed by the tertiary industry and the primary industry, and the average annual decline rates were 4.47% and 1.73%, respectively. Thus, the secondary industry was the greatest mitigation effect because energy intensity presented the biggest declining rate. Besides, the energy intensity of the secondary industry has been basically maintained the same changes as the whole industry (Figure 5b). The energy intensity of secondary industry should be further reduced in the future.

#### 4.2.2. Economic Output

It can be seen from Figure 4 that the economic output effect has always played a key driving role in increasing CO_2_ emissions, and the additive decomposition effect was 2.44 × 10^8^ t with the cumulative contribution rate of 201.94% during 2000–2017. Therefore, economic output was the most prominent determinant effect on CO_2_ emissions growth, which was in line with the various previous studies at the national level [15,53,54]. The fundamental reason is that the economic development is still at low-level stage in Gansu. Economic development is the necessary condition to satisfy the basic needs of people’s material life and cannot be separated from energy consumption. The economic development leads to the increase of energy consumption and then results in the growth of CO_2_ emissions, this is an unavoidable trend [55]. Gansu’s GDP has increased from 127.18 × 10^8^ USD in 2000 to 821.63 × 10^8^ USD in 2017 (Figure 3a), with its annual average growth rate of 11.60%. In the same period, the CO_2_ emissions have increased from 7805.70 × 10^4^ t to 19,896.05 × 10^4^ t (Figure 3b), with its annual average growth rate of 5.66%. Therefore, it could be seen that the increasing trend of CO_2_ emissions was roughly consistent with the economic output.

The economic output effect of each industry played the driving role in CO_2_ emissions growth during 2000–2017. As can be seen from Figure 5a, the cumulative contribution value of economic output in the secondary industry was 22,771.82 × 10^4^ t, followed by the tertiary and then by the primary industry with the contribution values of 1346.31 × 10^4^ t and 294.92 × 10^4^ t, respectively. It could be found that the secondary industry was the main sub-industry in increasing the effect of economic output with the contribution rate of 93.28%, and the GDP of secondary industry accounted for 44.57% in total GDP. In contrast, the contribution rate of the tertiary industry to the economic output effect was merely 5.51%, while its GDP accounted for 44.16% in total GDP. This was consistent with the characteristics of high energy consumption, high pollution, and high CO_2_ emissions in the secondary industry. At the same time, the tertiary industry showed the characteristics of low energy consumption, low pollution, and high output. Besides, the changes of secondary industry’s economic output had a crucial influence on the total CO_2_ emissions. Therefore, the proportion of the secondary industry should be gradually reduced, whereas the tertiary industry should be increased to mitigate Gansu’s CO_2_ emissions.

#### 4.2.3. Industrial Structure

**Figure 6:**
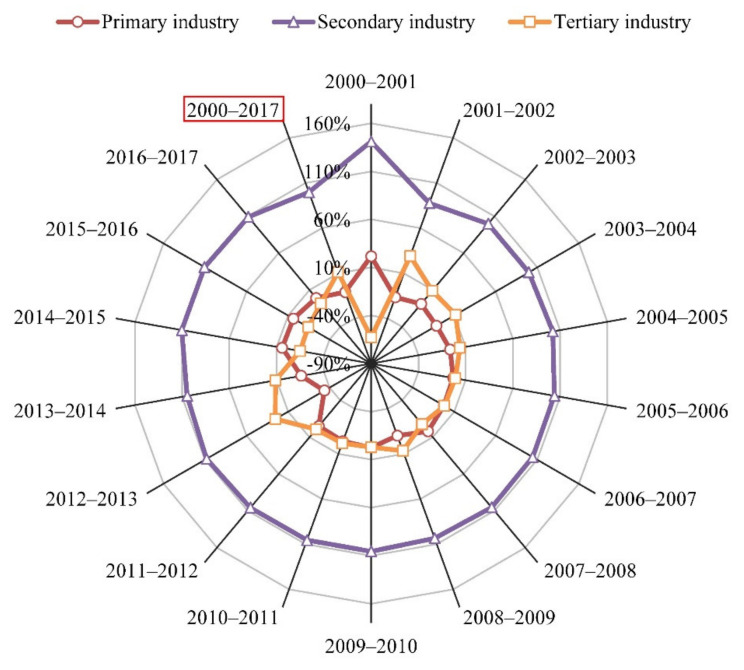
The proportions of industrial structure effect of each industry in Gansu from 2000 to 2017 and the entire period.

The additive decomposition effect of the industrial structure was 0.12 × 10^8^ t (Figure 4), and the cumulative contribution rate was 9.68% during 2000–2017. In total, the industrial structure was the second most significant driver after economic output, which was similar to the results provided by Zhang et al. [53], who illustrated that there existed some promoting effect of industrial structure on CO_2_ emissions increase. The effect of industrial structure failed to mitigate the CO_2_ emissions growth. The primary reason was that the driving effect of the secondary industry accounted for the largest proportion in industrial structure, and the proportion was invariably more than 85% from 2000 to 2017 (Figure 6). The secondary industry accounted for 99.96% in the effect of the industrial structure during 2000–2017, followed by the tertiary and the primary industry which accounted for 11.22% and −10.88%, respectively (Figure 6). The driving effect of the secondary industry offsets the mitigation effect of the primary industry for CO_2_ emissions growth.

The cumulative contribution values of industrial structure in the three different industries were −127.38 × 10^4^ t, 1166.52 × 10^4^ t, and 131.35 × 10^4^ t, respectively (Figure 5a). The primary industry presented the mitigation effect possibly for the reason that its internal structure was relatively reasonable. The climatic conditions of Gansu are suitable for the development of agriculture, animal husbandry, services in support of agriculture, but are unsuitable for the development of forestry and fishery. The proportions of agriculture, animal husbandry, services in support of agriculture, forestry, and fishery were 68.20%, 18.59%, 10.30%, 2.74%, and 0.17%, respectively. In contrast, both the secondary industry and the tertiary industry played driving effects because the internal structure of sub-industries was relatively unreasonable. In a word, the absolute contribution value of the industrial structure was relatively higher among the four driving effects. These results indicate that the industrial structure of Gansu will need to be further optimized in the future. Thus, the mitigation effect of the primary industry should be unceasingly enhanced. At the same time, both the secondary industry and the tertiary industry should be constantly changed from presenting driving effect to presenting mitigation effect.

#### 4.2.4. Population Scale

**Figure 7:**
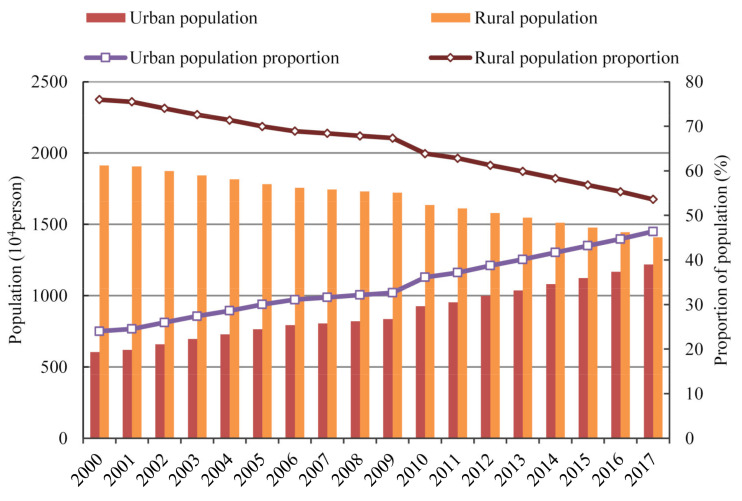
Changes in the number and the proportion of urban and rural population in Gansu Province from 2000 to 2017.

The effect of population scale was a driver of CO_2_ emissions growth from 2000–2017, with the additive decomposition effect of 0.09 × 10^8^ t and the cumulative contribution rate of 7.85% (Figure 4). The result of this study was in line with the results of an array of previous literature [15,54,56]. The population scale showed a driving effect because Gansu’s urban population presented an upward growth trend. As shown in Figure 7, it was observed that the rural population decreased from 1911.38 × 10^4^ persons in 2000 to 1407.64 × 10^4^ persons in 2017. However, the urban population increased from 603.93 × 10^4^ persons in 2000 to 1218.07 × 10^4^ persons in 2017, and the proportion of urban population increased by 22.83% during 2000–2017. With the rise of urban population, the level of urbanization has been constantly improved and the process of urbanization has been also accelerated. These promoted the development of industry, transportation, construction, and service industry. Moreover, the construction of urban infrastructure has been accelerated expansion. These led to the continuous extension of energy demand, which in turn brought about the increase of energy consumption, and then resulted in the growth of CO_2_ emissions. In addition, the secondary industry played the most important role in the effect of population scale with the cumulative contribution value of 889.44 × 10^4^ t during 2000–2017, followed by the tertiary industry and then by the primary industry with the cumulative contribution values of 51.1 × 10^4^ t and 6.21 × 10^4^ t, respectively (Figure 5a).

#### 4.2.5. Energy Structure

**Figure 8:**
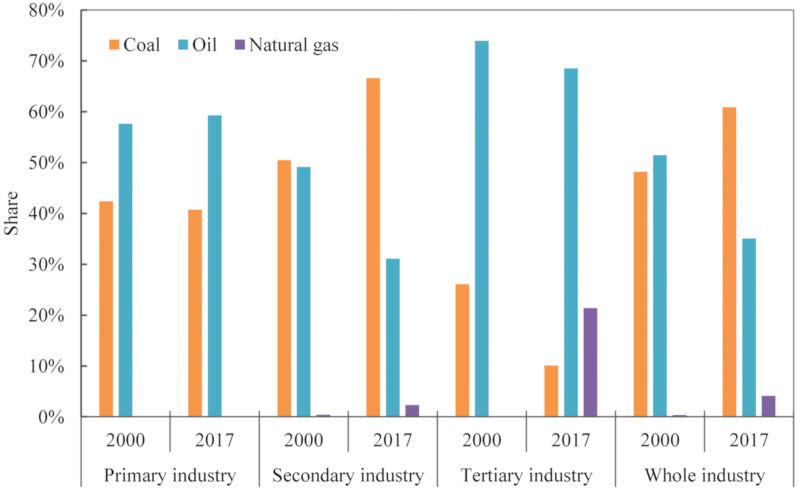
The energy consumption structure of each industry in 2000 and 2017.

As shown in Figure 4, the effect of the energy structure had the least impact on CO_2_ emissions growth during 2000–2017 when compared with other driving effects. The total additive decomposition effect of energy structure was only 0.04 × 10^8^ t with the cumulative contribution rate of 3.05%. These findings were consistent with Ma et al. [57], which indicated that the contribution of energy structure effect on China’s CO_2_ emissions from energy consumption was positive. From the energy consumption structure of the whole industry, it was noteworthy that the share of coal presented a moderate upward trend and increased from 48.22% in 2000 to 60.88% in 2017 (Figure 8), which accounted for the largest proportion in the total of energy consumption. Therefore, the energy consumption structure of Gansu had not changed too much. It had maintained a high-carbon energy consumption structure dominated by coal, and the share of natural gas was only 4.07% in 2017 (Figure 8). This might explain the reason why the effect of energy structure showed the driving effect. Overall, the optimization of energy structure must not be neglected in the CO_2_ reduction of Gansu.

The cumulative contribution values of energy structure in the primary industry and the secondary industry on the CO_2_ emissions were 3.92 × 10^4^ t and 462.33 × 10^4^ t in 2000–2017 (Figure 5a). The above two sub-industries showed driving effects because the energy consumption structure within the industry was relatively unreasonable. As shown in Figure 8, it was observed that the shares of coal and oil in the primary industry were 40.74% and 59.26% in 2017. At the same time, the share of coal in the secondary industry presented an upward trend and increased from 50.47% in 2000 to 66.71% in 2017 (Figure 8). It ranked first, followed by oil and natural gas. Both the primary and the secondary industry have maintained the high-carbon energy consumption structure dominated by coal and oil. In addition, the cumulative contribution value of energy structure in the tertiary industry was −97.12 × 10^4^ t during 2000–2017 (Figure 5a). The tertiary industry showed a mitigation effect because the energy consumption structure was relatively reasonable. To be specific, the share of coal presented a downward trend and declined from 26.05% in 2000 to 10.10% in 2017, whereas the share of natural gas increased from zero to 21.39% between 2000 and 2017 in the tertiary industry (Figure 8). Therefore, the adjustment of energy consumption structure cannot be ignored in Gansu, especially in the primary industry and the secondary industry.

### 4.3. Two-Dimensional Decoupling Analysis

Based on the GDP per capita and the per capita CO_2_ emissions of Gansu during 2000–2017, this paper empirically tested the EKC hypothesis of CO_2_ emissions by Eviews10 and estimated the regression coefficient of EKC. The regression analysis results were listed in Table 4, and the curve equation can be expressed as:

(13) $$C_{t}=0.86+51.87g^{t}-95.20{\left(g^{t}\right)}^{2}$$

The results of regression showed that $\alpha_{1}$ > 0, $\alpha_{2}$ < 0, so the EKC curve of GDP per capita and the per capita CO_2_ emissions demonstrated the inverted U-shape, which was in line with the Hao et al. [58], indicating that the relationship between carbon emissions and GDP per capita was an inverted U-shape here in China. The significance probability value (Prob.) was less than 10%, which indicated that the coefficient was marked. The R-square was 0.972 and was close to 1, which indicated that the goodness of fit was high. In addition, the threshold of GDP per capita (g*) was 0.2724 × 10^4^ USD.

**Figure 9:**
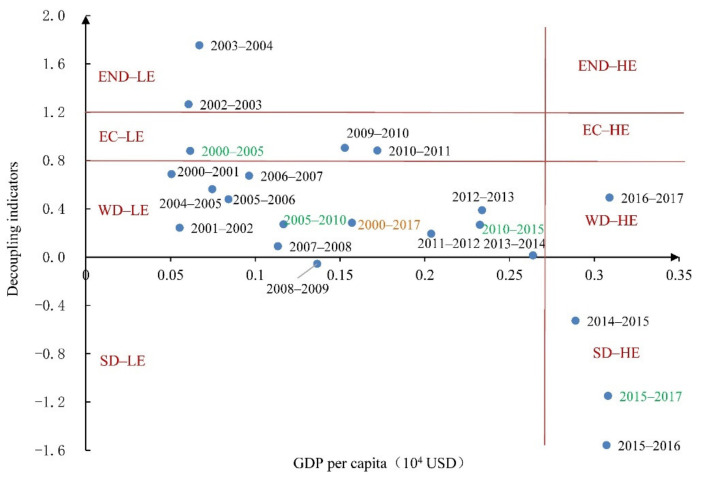
Results of two-dimensional decoupling analysis between economic development and CO_2_ emissions in Gansu from 2000 to 2017 and four stages (2000–2005, 2005–2010, 2010–2015, and 2015–2017).

The results of the two-dimensional decoupling status between CO_2_ emissions and the economic growth in Gansu were listed in Figure 9 and showed a low-level weak decoupling (WD-LE) during 2000–2017. In other words, the rapid economic growth has been accompanied by the relatively higher growth rate of CO_2_ emissions, while the growth rate of CO_2_ emissions was smaller than the economic growth rate. Besides, Gansu’s economy had experienced a low level of economic development (LE) in 2000–2014 because the GDP per capita was smaller than the threshold value of GDP per capita (Figure 9). In contrast, it had experienced a high level of economic development (HE) in 2014–2017 (Figure 9). Furthermore, the two-dimensional decoupling status tended to be unstable. Six states occurred in Gansu during 2000–2017 (Figure 9), namely, WD-LE (accounting for 52.94% of the entire study period), low level-expansion negative decoupling (END-LE), low level-expansion coupling (EC-LE), low level-strong decoupling (SD-LE), high level-strong decoupling (SD-HE), and high level-weak decoupling (WD-HE). In order to better understand the underlying mechanism, this paper analyzed the changes of the two-dimensional decoupling status in four different stages (2000–2005, 2005–2010, 2010–2015, and 2015–2017).

During stage 1 (2000–2005), the two-dimensional decoupling state was EC-LE (Figure 9). The economic development was at the low-level stage, with the GDP per capita of 0.06 × 10^4^ USD and the decoupling indicator of 0.88. This could be due to the fact that the Western Development Strategy has been implemented to support the development of western regions since 2000. At the same time, the various advantageous resources were continuously exploited, and many enterprises with high energy consumption, high pollution, and high CO_2_ emissions were established to develop Gansu’s economy. Moreover, ten major engineering projects were launched in Gansu to carry out infrastructure construction. These made GDP and CO_2_ emissions increase by 68% and 60%, respectively. In other words, economic development and CO_2_ emissions have maintained relatively synchronous growth. Furthermore, the driving force of economic development was sufficient, and the environmental pollution was serious.

During stage 2 (2005–2010), the two-dimensional decoupling state was WD-LE (Figure 9). The GDP per capita was 0.12 × 10^4^ USD, and the decoupling indicator was 0.27 during this stage. The economic growth rate peaked at 105.66%, while the growth rate of CO_2_ emissions was relatively small at 35%. This might be explained by the fact that the major projects of environmental governance were implemented, and the management of energy consumption in key enterprises was strengthened by the Gansu government. Meantime, cleaner production and low-carbon development of industry were continuously promoted in Gansu. Hence, the CO_2_ emissions intensity of the whole industry declined from 58.38 t/10^4^ USD in 2005 to 38.33 t/10^4^ USD in 2010, and the energy intensity declined from 23.42 tsce/10^4^ USD in 2005 to 14.96 tsce/10^4^ USD in 2010 (Figure 5b). Therefore, the economic development and CO_2_ emissions showed a weak decoupling state.

During stage 3 (2010–2015), the two-dimensional decoupling state was also WD-LE (Figure 9). The GDP per capita was 0.23 × 10^4^ USD, which was higher than those at the previous stages. The decoupling indicator dropped to 0.267, and the weak decoupling status was more ideal than those at the previous stages. The growth rates of GDP and CO_2_ emissions were 81% and 22%, respectively. This major reason was that a new round of Western Development Strategy has been implemented since 2010 and the Silk Road Economic Belt has been proposed since 2013. Meanwhile, a few key projects in transportation, industry, and energy have been established in Gansu. As a result, the CO_2_ emissions intensity and energy intensity have been further reduced to 25.73 t/10^4^ USD and 10.03 tsce/10^4^ USD, respectively. The comprehensive economic strength was enhanced in Gansu. Therefore, the decoupling state at this stage was more ideal than those at the previous stages.

During stage 4 (2015–2017), the two-dimensional decoupling state changed from WD-LE to SD-HE (Figure 9). The GDP per capita was 0.31 × 10^4^ USD and was bigger than the threshold value of GDP per capita. The decoupling indicator decreased to −1.15 and the average annual growth rate of CO_2_ emissions decreased to 1.67%. This could be explained by the fact that the total amount of energy consumption was controlled, and the technology of enterprise production was strengthened to further improve the energy efficiency of Gansu. As a result, CO_2_ emissions intensity and energy intensity have decreased to 24.35 t/10^4^ USD and 9.55 tsce/10^4^ USD during this stage, respectively. The decoupling status was SD-HE in this stage, but this didn’t mean that the economic development has reached the high-level stage and the decoupling state has reached strong decoupling in Gansu because it must take a long time to achieve SD-HE.

## 5. Conclusions and Policy Recommendations

### 5.1. Main Conclusions

Based on the data of energy consumption in Gansu during 2000–2017, we calculated the changes of Gansu’s CO_2_ emissions by applying the IPCC carbon emissions accounting method and decomposed the corresponding drivers by using the LMDI model. The two-dimensional decoupling model was used to analyze the decoupling relationship between CO_2_ emissions and economic development. The main conclusions were acquired as follows.

From the perspective of Gansu’s CO_2_ emissions, the total amount of CO_2_ emissions increased from 7805.70 × 10^4^ t in 2000 to 19,896.05 × 10^4^ t in 2017, with its average annual growth rate being 5.66%. As a whole, the total CO_2_ emissions in Gansu showed an upward trend in fluctuation with the fluctuating increase of energy consumption. It presented an upward trend between 2000–2014 and a downward trend from 2014–2017. In terms of the industry sources of CO_2_ emissions, the secondary industry received the lion’s share, accounting for 92% of the total CO_2_ emissions, followed by the tertiary industry and the primary industry with the proportions of 5.90% and 1.21%, respectively.

From the perspective of the decomposition effects analysis in Gansu province, the cumulative contributions of energy structure, energy intensity, industrial structure, economic output, and population scale were 0.04 × 10^8^ t, −1.48 × 10^8^ t, 0.12 × 10^8^ t, 2.44 × 10^8^ t, and 0.09 × 10^8^ t, respectively. These results indicated that the growth of economic output was the most important driving effect of CO_2_ emissions growth followed by industrial structure, population scale, and energy structure, and their cumulative contribution rates were 201.94%, 9.68%, 7.81%, and 3.05%. However, energy intensity exhibited the maximum mitigation effect, and the cumulative contribution rate was −122.49%. Moreover, the industrial structure and energy structure of the three sub-industries had different effects on CO_2_ emissions. The industrial structure factor of the primary industry and the energy structure factor of the tertiary industry had mitigation effect on CO_2_ emissions growth. The second industry took the largest proportion in terms of the sub-industries’ contribution as regards the decomposition effects.

From the perspective of the two-dimensional decoupling analysis, the EKC curve of GDP per capita and the per capita CO_2_ emissions in Gansu was demonstrated an inverted U-shape. The two-dimensional decoupling status between CO_2_ emissions and economic growth was WD-LE in Gansu during 2000–2017. The economic development was in a low-level development stage and the decoupling state was in weak decoupling. The changes of the two-dimensional decoupling status were in four different stages, namely, EC-LE, WD-LE, WD-LE, and SD-HE, respectively. In addition, the tendency of two-dimensional decoupling states was unstable, and there were six states over the study period, while the WD-LE accounted for 52.94% during the entire study period. These results show that it will need to take a long time for Gansu to achieve the SD-HE status.

### 5.2. Policy Recommendations

According to the conclusions drawn above, in order to reduce CO_2_ emissions of Gansu and achieve the goals of carbon peak by 2030 and carbon neutral by 2060, some policy recommendations are put forward as follows for Gansu government’s possible reference.

First, in accordance with the empirical results, the CO_2_ emissions of energy consumption in the secondary industry accounted for the maximum proportion, followed by the tertiary industry and then by the primary industry. Therefore, it should be the focus of CO_2_ emissions reduction policy to optimize the industrial structure in Gansu. The secondary industry is the main sector for CO_2_ emissions reduction. It is important and necessary for the Gansu government to eliminate the traditional enterprises with backward technology in the secondary industry and levy environmental tax and energy tax. Furthermore, the sub-industries of industry, transportation, storage, and post are the key sectors of emission reduction in the future. Meanwhile, the development of low-carbon enterprises should be encouraged and supported, such as the sub-industries of electronic information, service, tourism, and commerce. In addition, Gansu is a typical agricultural province. It is essential to stabilize the development of agriculture, take the road of developing agriculture by science and technology, and even build a strong province of animal husbandry.

Second, the effect of economic output was the biggest driving factor in CO_2_ emissions growth mentioned above. However, Gansu is located in western regions with abundant energy and enjoys the huge potential for further development. It is not feasible to reduce CO_2_ emissions by curbing economic development. Therefore, the Gansu government should improve the quality and efficiency of economic development. It is suggested that when introducing investments from different industries into Gansu through the “Silk Road Economic Belt” strategy, two points, namely, high-tech industries and low-carbon industries, should be the key sections through which to effectively mitigate CO_2_ growth. In addition, as noted above, the cumulative contribution of the energy structure was positive. Thus, the Gansu government ought to adjust the energy structure by dropping coal consumption especially in the secondary industry, improving coal use efficiency and increasing the utilization of clean coal technologies. Furthermore, the effect of energy intensity was the largest mitigation factor in CO_2_ emissions. Thus, it is necessary for Gansu government to further decrease energy intensity. Some feasible ways might include making innovations of low-carbon technology and introducing advanced technologies from developed regions both home and abroad.

Finally, considering the two-dimensional decoupling state was WD-LE in Gansu during 2000–2017, Gansu government might need to boost a sustainable economic development to realize the SD-HE between CO_2_ emissions reduction and economic development. Thus, it is imperative for Gansu government to formulate the policies of energy-saving and emission-reduction according to the actual situation of economic development level. Meanwhile, all kinds of energy should be utilized in a circular pattern as far as possible to improve energy utilization efficiency. The investment of new energy should be strengthened in Gansu, such as hydropower, wind power, solar energy, biomass energy, and geothermal energy, to name but a few, to further reduce the decoupling coefficient.

### 5.3. Limitations and Further Perspectives

Although some meaningful conclusions have been drawn in this paper, there are still some limitations, which could also provide us with some hints about our possible future research directions. First, merely five influencing factors were explored, which affected the energy-related CO_2_ emissions of the three different industries in Gansu, specifically economic output, energy intensity, industrial structure, population size, and energy structure. However, carbon emissions intensity, R&D efficiency, R&D intensity, and investment intensity, among other things, may also exert some effect on CO_2_ emissions. Thus, attention should be given to these influencing factors left out in Gansu CO_2_ emissions in our future endeavor. Second, this paper only analyzed the two-dimensional decoupling state, without combining the complete decomposition model with the decoupling index to carry out an attribution analysis. Therefore, in the near and foreseeable future, it is quite necessary for us to conduct a comprehensive decoupling analysis between Gansu’s CO_2_ emissions and its economic development by quantifying the decoupling status, exploring the factors affecting decoupling, and measuring the effectiveness of decoupling efforts.

## Figures and Tables

**Table 1:** CO_2_ emissions coefficients of different energy types.

Energy Type	Carbon Content (kg-CO_2_/GJ)	Carbon Oxidation (%)	Net Calorific Value (TJ/Gg)	Emission Coefficient (CO_2_/t)
Coal	26.0	100	20.9	1.993
Coke	29.2	100	28.2	3.044
Crude Oil	20.0	100	42.3	3.102
Gasoline	20.2	100	43	3.185
Kerosene	19.50	100	44.1	3.153
Diesel Oil	20.2	100	43	3.185
Fuel Oil	21.1	100	40.4	3.126
Liquefied Petroleum Gas	17.20	100	47.3	2.983
Natural Gas	15.32	100	38931 *	21.840 **

Note: * The unit is KJ/m^3^, ** The unit is t-CO_2_/10^4^ m^3^.

**Table 2:** The definition of variables.

Variable	Definition
$C_{ij}$	CO_2_ emissions in industry i based on j fuels
$E_{i}$	Energy consumption of the industrial i
$G_{i}$	GDP of the industrial i
G	GDP of Gansu
P	Total population of Gansu province
$F_{ij}$	CO_2_ emissions coefficient of energy j
$S_{ij}$	Share of energy j in industry i
$I_{i}$	Energy consumption per unit of GDP in industry i
$D_{i}$	Share of industry i in total GDP
R	GDP per unit of population

**Table 3:** Judgment criteria of the Tapio decoupling model.

Decoupling State	ΔC/C	ΔGDP/GDP	D
Expansive negative decoupling (END)	>0	>0	(1.2, +∞)
Strong negative decoupling (SND)	>0	<0	(−∞, 0)
Weak negative decoupling (WND)	<0	<0	[0, 0.8)
Weak decoupling (WD)	>0	>0	[0, 0.8)
Strong decoupling (SD)	<0	>0	(−∞, 0)
Recessive decoupling (RD)	<0	<0	(1.2, +∞)
Expansive coupling (EC)	>0	>0	[0.8, 1.2]
Recessive coupling (RC)	<0	<0	[0.8, 1.2]

**Table 4:** Regression results of EKC curve.

Variable	Coefficient	Std. Error	t-Statistic	Prob.
$\alpha_{0}$	0.864912	0.337022	2.566336	0.0215
$\alpha_{1}$	56.86681	4.657245	11.13680	0.0000
$\alpha_{2}$	−95.20390	12.62758	−7.539362	0.0000

## Data Availability

The data presented in this study are available on request from the corresponding author.
